# Two new species of *Ardisia* subgenus *Tetrardisia* (Primulaceae-Myrsinoideae) from Borneo

**DOI:** 10.3897/phytokeys.145.48573

**Published:** 2020-04-10

**Authors:** Avelinah Julius, Tadashi Kajita, Timothy M.A. Utteridge

**Affiliations:** 1 Forest Research Institute Malaysia, Kepong, Selangor, 52109, Malaysia Forest Research Institute Malaysia Kepong Malaysia; 2 Department of Biology, Graduate School of Science, Chiba University, 1-33 Yayoi-cho, Inage-ku, Chiba, 263-8522, Japan Chiba University Chiba Japan; 3 Iriomote Station, Tropical Biosphere Research Center, University of the Ryukyus, 870 Uehara, Taketomi-cho, Yaeyama-gun, Okinawa, 907-1541, Japan University of the Ryukyus Okinawa Japan; 4 United Graduate School of Agricultural Science, Kagoshima University, 1-21-24 Korimoto, Kagoshima, 890-0065, Japan Kagoshima University Kagoshima Japan; 5 Identification & Naming Department, Royal Botanic Gardens, Kew, Richmond, Surrey, TW9 3AE, UK Royal Botanic Gardens London United Kingdom

**Keywords:** Conservation, endemic, key, Malesia, Myrsinaceae, South-East Asia, taxonomy

## Abstract

*
Ardisia argentiana* and *A. nagaensis* from subgenus Tetrardisia are herein described and illustrated as new species. They are endemic to Borneo and the Indonesian province of Central Kalimantan and to the Malaysian state of Sarawak, respectively. *Ardisia argentiana* is unique in its linear-oblong leaves, with a long, acuminate-caudate apex, and finely serrulate margins, while *A. nagaensis* can be easily recognized by its elliptic-lanceolate leaves.

## Introduction

*Ardisia* Sw. with ca. 450 to over 1000 species (Stevens 2001 onwards; Frodin 2004), is the largest tropical genus in subfamily Myrsinoideae of the enlarged Primulaceae (as circumscribed by the Angiosperm Phylogeny Group, APG 2009). The genus was last revised by Mez (1902) within his monograph of the Myrsinaceae, but there are no modern species level treatments. In South-East Asia, contemporary work on the genus has resulted in publication of several species (e.g. Stone 1989a, Stone 1990; Julius et al. 2017), and regional accounts for New Guinea (Sleumer 1988) and Peninsular Malaysia (as an annotated key in Stone 1989a). For Borneo, there is no recent detailed treatment of the genus from the island, but Stone (1989b) published a provisional checklist of *Ardisia* with 80 species, and an additional 14 species were added to these by Hu (2002: 13 species) and Utteridge et al. (2014: 1 species).

*Ardisia* is classified into subgenera on characters of habit, leaf morphology, disposition of flowers at inflorescence branch apices (racemes, umbels, corymbs), inflorescence position and floral morphology (see Mez 1902), and the two new species described here are members of subgenus Tetrardisia (Mez) K. Larsen & C.M. Hu (Larsen and Hu 1995). Originally described as a monotypic genus by Mez (1902), based on the four-merous corolla and the low ovule number, *Tetrardisia* was reduced to a subgenus of *Ardisia* by Larsen and Hu (1995) due to a better understanding of character variation within *Ardisia* - both merosity and ovule number are now known to vary within previously described subgenera. Ståhl and Anderberg (2004) subsequently recognized it as a valid genus, using characters of merosity and ovules in their key to genera but did not discuss this opinion noting only that *Tetrardisia* was “treated as subgenus of *Ardisia* by Larsen and Hu (1995).” Initial molecular findings, however, have shown that species of *Tetrardisia* are nested within *Ardisia* (Julius et al. 2016; Julius 2019), and the classification of Larsen and Hu (1995) is followed here.

The subgenus is defined by the combination of the small, woody shrub habit, leaves lacking bacterial nodules with a finely serrulate-denticulate leaf margin, and the usually tetramerous flowers with few ovules. During study of *Ardisia* specimens from Borneo deposited at KEP and E, two unidentified specimens with tetramerous flowers were encountered and suspected to be undescribed taxa. After close scrutiny of the relevant literature (e.g. Furtado 1959; Larsen and Hu 1991; Stone 1992), and comparison with all other *Tetrardisia* species, we found the specimens do not match any of those known species. Thus, the unidentified specimens collected from Kalimantan and Sarawak are apparently new and described here as new species.

There are very few species of *Ardisia* with tetramerous flowers, and with few occurring in Malesia and surrounding regions: only a single taxon is recognized each from Java and Thailand, and three are known from Peninsular Malaysia, with none currently described from Borneo. With the publication of the two new species here, subgenus Tetrardisia now has six species, the previously described species, *A. denticulata* Blume (Blume 1826: 691), *A. porosa* C.B. Clarke (Clark 1882), *A. tetramera* K. Larsen & C.M. Hu (Larsen and Hu 1991), *A. tetrasepala* King & Gamble (King and Gamble 1905, plus the new species herein: *A. argentiana* Julius & Utteridge sp. nov. and *A. nagaensis* Julius, T. Kajita & Utteridge sp. nov.

## Materials and methods

This study was based on examination of herbarium specimens at E, K and KEP and the relevant taxonomic literature (e.g. Furtado 1959; Larsen and Hu 1991; Stone 1992); in addition, specimen images from Global Plants JSTOR (http://plants.jstor.org/) and the BioPortal of Naturalis Biodiversity Center (http://bioportal.naturalis.nl/) were consulted. Herbaria are abbreviated according to the Index Herbariorum (Thiers 2016). All measurements were taken from herbarium specimens and rehydrated material for floral description, shape terminology follows Systematics Association Committee (1962). The conservation status was assessed using the IUCN criteria (IUCN 2012).

## Taxonomic treatment

### Ardisia argentiana

Taxon classificationPlantaeEricalesPrimulaceae

Julius & Utteridge sp. nov.

B659B9AB-AF97-59E7-92C7-553D71C2FEA3

urn:lsid:ipni.org:names:77209338-1

Figure 1

#### Diagnosis.

Differs from other members of the subgenus Tetrardisia in having linear-oblong leaves, with a long, acuminate-caudate apex, and finely serrulate margins.

#### Type.

INDONESIA. Borneo: Central Kalimantan, Kotawaringan [Kotawaringin] Timur, S. Mentaya, km 92 from Sangai, Plot 8, [1°18’S, 112°32’E], 100 m elevation, 18 May 1993, *Argent et al. 93187* (holotype E!; iso: BO).

**Figure 1.**
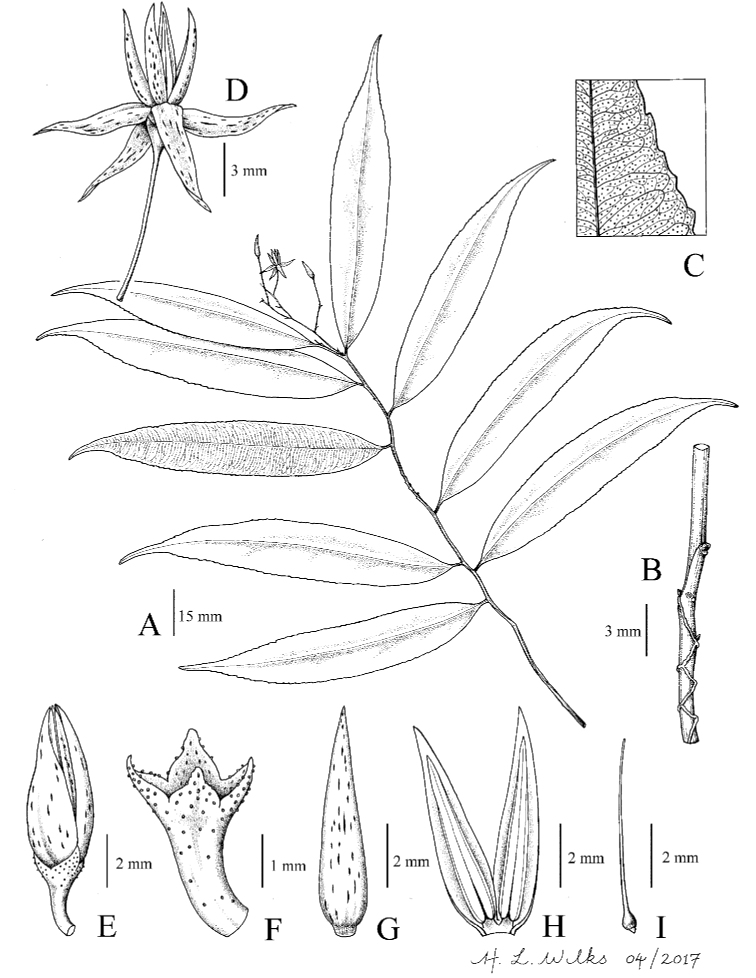
*Ardisia argentiana* Julius & Utteridge, sp. nov. **A** habit **B** stem with flexuose line **C** venation details on lower surface **D** mature flower **E** flower bud **F** calyx **G** corolla lobe, abaxial view **H** open corolla and stamen **I** ovary and pistil. (Drawn by Hazel Wilks from *Argent et al. 93187*).

#### Description.

Shrub ca. 1 m high; stems sparsely scaly when young, soon glabrous, slightly flexuous, winged between the nodes with raised lines running along the internodes between the petiole bases. *Indumentum* of sessile, circular, peltate scales up to 0.05 mm in diameter, on young parts, leaves and inflorescence. *Leaves* alternate; petioles 3–5 mm long, winged by the decurrent leaf base, glabrous; lamina chartaceous, linear-oblong, (7–)10–11.5 × 1–2 cm, with dense gland-dots throughout apex acuminate-caudate, acumen 1–2.5 cm long; base cuneate, between the higher order venation, lacking hairs, sparsely scaly on the lower midrib, glabrous above, midrib sunken above, raised beneath; lateral veins ca. 48 pairs, semicraspedodromus, and with 1–2 intersecondary veins within each pair; intercostal veins obscure. *Inflorescences* terminal on lateral branches, proximally laxly paniculate with subumbellate branches with 2-flowers distally racemose; peduncle and rachis (1.5–)2.5–4 cm long, slightly flexuose, sparsely scaly; bracts linear-lanceolate, (0.4–)1.5–1.7 × 0.4 mm, very sparsely ciliate with few scattered hairs. *Flowers* ca. 8; pedicels slender, ca. 1.5 cm long, sparsely scaly; calyx-lobes 4, green, gland-dotted, lacking hairs, sparsely scaly outside, ovate, ca. 1 × 0.8 mm, apex acute, margin very sparsely ciliate with few scattered short hairs; corolla-lobes 4, reflexed, twisted apically, white with orange-brown elongated-dots, lanceolate, 6.4–7 × 1.7–2.3 mm, glabrous on both surfaces; stamens 4, spreading upright (in open flower), filament ±sessile, anthers twisted apically, lanceolate, ca. 5–6.2 × 0.8 mm, apex elongated into a hyaline tip, thecae open by longitudinal-slits, with scattered, lineate, orange brown dots behind, glabrous; ovary ovoid, ca. 7 × 6 mm, style and stigma filiform, ca. 6.8 mm long, ovules ca. 6 in 1-series. *Fruits* n.v.

#### Distribution.

Endemic to Borneo; known only from Sungai Mentaya, Kotawaringin Timur, Central Kalimantan.

#### Etymology.

The species epithet commemorates Graham Charles George Argent (1941–2019), a prominent botanist on tropical botany in South-East Asia and a leading expert on Ericaceae, especially the tropical ‘Vireya’ rhododendrons, and collector of the type material.

#### Conservation status.

Data deficient (DD). The only specimen available was collected in 1993 and the species is known only from a single collection location south of Bukit Raya in Central Kalimantan, and thus meets the B1a criterion for Critically Endangered (CR) status. The species was found to the south of the Bukit Baka – Bukit Raya National Park, and, to date, there are no further collections of the species from inside this protected area. Satellite imagery in Google Earth from 2015 shows that the collection locality still has some forest coverage and was not penetrated with roads or logging tracks, and it also appears to not have been converted for agricultural use such as oil palm plantations. However, lack of collections and field observations of the species do not allow inference of decline or fluctuation in population size or EOO and AOO, and we are unable to fulfil the criteria to preliminary assess this species as Critically Endangered.

#### Notes.

*Ardisia argentiana* is a distinct species on account of the combination of branches with wing-like raised lines running between the petiole bases, linear-oblong leaves less than 2 cm wide with finely serrulate margins, the terminal, laxly paniculate inflorescence with a hairy scaly rachis and few, tetramerous flowers. This new species is morphologically similar to *Ardisia nagaensis*, but differs from that species in leaf morphology, especially the shorter pedicels (*A. argentiana* 3–5 mm long; *A. nagaensis* 1.5– 3 cm long), linear-oblong leaves less than 2 cm wide (*A. nagaensis* elliptic-lanceolate to 5.5 cm wide) and the fewer flowered inflorescence (*A. argentiana* with 8 flowers vs. *A. nagaensis* with 24).

The flower is described as ‘white’ in the specimen label which probably refers to the corolla and anthers. The leaf resembles *Ardisia mystica*. B.C. Stone (Stone 1982), a member of subgenus Pimelandra, but all members of that subgenus have short, axillary inflorescences. Similar wing-like ridges along the internodes are found in species of *Systellantha*, a genus of three species endemic to northern Borneo (Drinkell and Utteridge 2015). Members of *Systellantha* are understorey small trees or shrubs to only 3 m, are also tetramerous but have unisexual flowers with the plants being monoecious. The wing-like ridges may be a response to ecological conditions, such as high humidity, in the rainforest understorey.

### Ardisia nagaensis

Taxon classificationPlantaeEricalesPrimulaceae

Julius, T. Kajita & Utteridge sp. nov.

2BA31A89-C364-5DCA-BCC3-2FA1601481A6

urn:lsid:ipni.org:names:77209339-1

Figure 2

#### Diagnosis.

Similar to *Ardisia tetrasepala* in its simple and compact inflorescence with corymbose flowers but differs mainly vegetatively in having leaves laxly arranged, longer petioles that are covered with glandular hairs, a chartaceous lamina without marginal secondary veins, surface with scattered stellate hairs near the margin beneath and leaf base obtuse or ±cuneate. The corolla lobes are smooth without lepidote scales (vs. lepidote scales present in *A. tetrasepala*).

#### Type.

MALAYSIA. Borneo: Sarawak, Tatau District, Ulu Merirai, Gua Naga, [02°39’12”N, 113°03’05”E], 11 July 2005, *Julia et al. S95726* (holotype KEP!; iso: SAR, SING)

**Figure 2.**
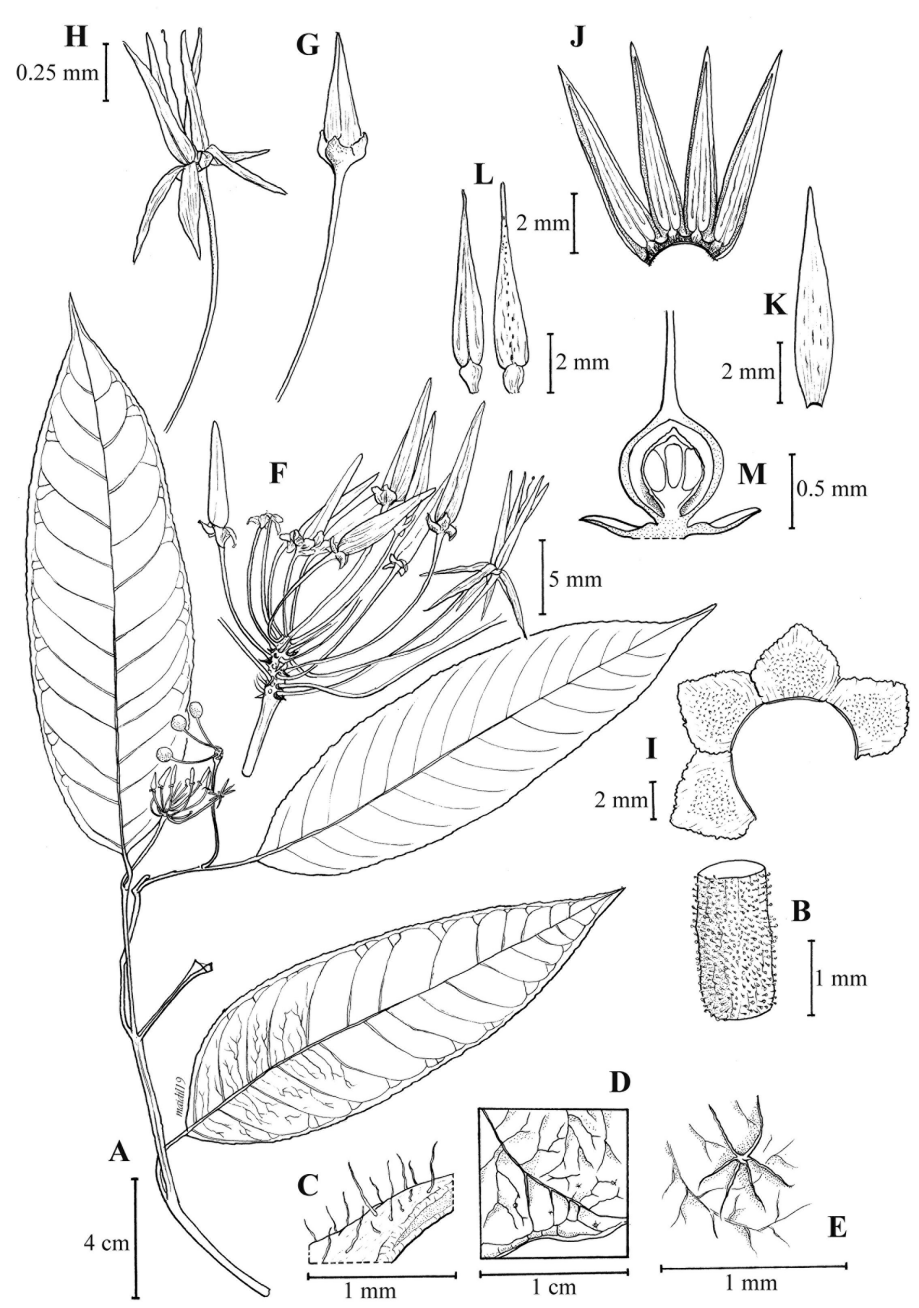
*Ardisia nagaensis* Julius, T. Kajita & Utteridge, sp. nov. **A** habit **B** glandular hairs on stem **C** erect, simple hairs on petiole **D** the venation close-up **E** stellate hairs close-up **F** enlarged inflorescence **G** flower bud **H** mature flower **I** open calyx **J** open corolla with stamen **K** corolla lobe **L** stamen, front and back view **M** placenta and ovules. (Drawn by Mohamad Aidil Noordin from *Julia et al. S95726*).

#### Description.

A small shrub, less than 1 m high; stems glabrescent, slightly flexuous, winged between the nodes with obscure raised lines running along the internodes between the petiole bases. *Indumentum* of simple, short (stalked), glandular, or stellate (star-liked), pale brown to rusty hairs (visible under microscope). *Leaves* alternate; petioles 1.5–3 cm long, sparsely to densely hairy towards midrib, simple, short, glandular hairs; lamina chartaceous, with dense gland-dots throughout the leaf, glabrous on both surfaces except for margin with scattered stellate hairs beneath, elliptic-lanceolate, 17–19 × 5–5.5 cm, apex long acuminate and slightly caudate with acumen 1.5–2.0 cm long; base obtuse or ±cuneate, margin obscurely denticulate being entire in appearance, midrib flat or slightly sunken above, raised beneath, glabrous except hairy with glandular hairs beneath, denser near leaf base; lateral veins 13–15 pairs, distinct above, prominent beneath, inter-secondary veins present in between; intercostal veins percurrent, distinct beneath. *Inflorescence* terminal on lateral branches, simple with flowers arranged in corymbs; peduncle and rachis 4–5 cm long, densely hairy with glandular hairs; bracts ovate to elliptic, 0.5–1.5 mm long, hairy with glandular hairs. *Flowers* ca. 24; pedicels slender, 1.2–1.8 cm long, up to 2 cm long in fruiting, covered with dense, glandular hairs; calyx-lobes 4, purplish, gland-dotted, lacking hairs, broadly ovate, 1–1.5 × 1–1.5 mm, apex obtuse, margin incised, sparsely ciliate with short, thick hairs; corolla-lobes 4, reflexed, twisted apically, purplish with transparent margin, lanceolate, ca. 8 × 2.5 mm, glabrous on both surfaces; stamens 4, spreading upright (in open flower), filament ±sessile, anthers twisted apically, lanceolate, ca. 6 × 1 mm, apex without a prolonged hyaline tip, thecae opening by longitudinal slits, with lineate, black dots behind, glabrous; ovary globose, ca. 1 × 0.8 mm, style and stigma filiform, ca. 6 mm long, ovules 4–6 in 1-series. *Fruits* ripe bright red, globose, ca. 6 × 5 mm.

#### Distribution.

Endemic in Borneo, Sarawak; known only from Gua Naga, Ulu Merirai area, Tatau District.

#### Etymology.

*Ardisia nagaensis* is very local and was found at only one locality, Gua Naga, for which it is named.

#### Conservation status.

Data deficient (DD). The only specimen available was collected in 2005 and the species is known only from a single collection location from Ulu Merirai in Central Sarawak, and thus meets the B1a criterion for Critically Endangered (CR) status. The Ulu Merirai region is an area of sandstone with limestone cliffs, and supports several point endemics, including several newly described species of *Begonia* L. (Kiew and Sang 2009) and *Monophyllea* R. Br. (Kiew and Sang 2013). The species was found outside of any protected area, but satellite imagery in Google Earth from 2017 shows that the collection locality has undisturbed forest coverage and was not penetrated with roads or logging tracks. However, lack of collections and field observations of the species do not allow inference of decline or fluctuation in population size or EOO and AOO, and we are unable to fulfil the criteria to preliminary assess this species as Critically Endangered.

#### Notes.

Compared to the other members of subgenus Tetrardisia, this new species described here has an affinity with *A. tetrasepala*, endemic to Peninsular Malaysia [Johor, Gunung Pulai]. This is because both are characterized by a simple and compact inflorescence, whereas other taxa in xTetrardisia have compound and laxly flowers arranged. *Ardisia nagaensis* differs from *A. tetrasepala* by the leaf characters viz. the petiole length (*A. nagaensis* with 1.5–3 cm long vs. *A. tetrasepala* with 0.5–0.8 cm long), the leaf base (*A. nagaensis* with obtuse or ±cuneate leaf base vs. *A. tetrasepala* with cordate-rounded) and the venation (*A. nagaensis* without marginal secondary veins vs. *A. tetrasepala* with marginal secondary veins). Further morphological comparison between these two species is given in the Table 1.

The calyx is described as ‘purplish’ in the specimen label which probably refers to both the calyx and corolla. The corolla margin on one side is transparent as observed in *A. denticulata*.

**Table 1.** Morphological comparison of *Ardisia nagaensis* and *A. tetrasepala*.

	***Ardisia nagaensis***	***A. tetrasepala***
Leaf texture	Chartaceous	Subcoriaceous
Leaf shape and size	Elliptic-lanceolate, 17–19 × 5–5.5 cm	Oblong-elliptic, 10–19.5 × 2.5–5 cm
Leaf base	Obtuse or ±cuneate	Cordate-rounded
Leaf margin	Obscurely denticulate being entire in appearance	Seemingly entire immature but finely crenulate when mature
Leaf apex	Long acuminate and slightly caudate with acumen 1.5–2.0 cm long	Acuminate-caudate, acumen 1.5–2.5 cm long
Leaf surface	Glabrous on both surfaces except for margin with scattered stellate hairs beneath	Glabrous above except sparsely hairy to glabrescent on midrib Beneath
Lateral veins	Lateral veins 13–15 pairs, distinct above, prominent beneath, inter-secondary veins present in between	Lateral veins 20–24, distinct and slightly prominent on both surfaces, brochidodromus with secondary veins looping and joining 0.2–0.5 mm in from the margin with distinct secondary loops
Intercostal veins	Percurrent, distinct beneath	Reticulate, distinct and slightly prominent on both surfaces
Flowers	ca. 24	8–13
Pedicel	12–18 mm long	5–10 mm long
Calyx lobes	Purplish, lacking hairs, broadly ovate, 1–1.5 × 1–1.5 mm	Green, glabrous inside, sparsely hairy outside, ovate, ca. 1 × 1 mm

### Key to species of Ardisia subgenus Tetrardisia

**Table:** 

1	Leaves entire or obscurely undulate or obscurely crenulate	**2**
–	Leaves distinctly crenulate-denticulate	**5**
2	Inflorescences paniculate, 15–20 cm long. Thailand and Malay Peninsula	***A. porosa***
–	Inflorescences subumbullate or racemose, 3–5 cm long	**3**
3	Leaf lamina linear-oblong, 1–2 cm wide, margin distinctly finely serrulate in the distal half. Inflorescence branched and flowers loosely arranged in racemes. Borneo	***A. argentiana***
–	Leaf lamina elliptic, elliptic-lanceolate or elliptic-oblong, 3.5–7 cm wide	**4**
4	Leaves with scattered stellate hairs near the margin abaxially, margin obscurely serrulate being entire in appearance; petioles 1.5–3 cm long. Inflorescence with ca. 24 flowers. Borneo	***A. nagaensis***
–	Leaves puberulent along the midrib abaxially, margins entire proximally becoming faintly crenulate distally; petioles 5–8 mm long. Inflorescences with 8–20 flowers. Malay Peninsula	***A. tetrasepala***
5	Leaves somewhat bullate, lateral nerves 12–20 pairs; pedicels puberulous. Malay Peninsula and Java	***A. denticulata***
–	Leaves not bullate, lateral nerves numerous; pedicels glabrous. Thailand	***A. tetramera***

## Supplementary Material

XML Treatment for Ardisia argentiana

XML Treatment for Ardisia nagaensis
